# The occurrence and human health risk assessment of total and aflatoxin B_1_ in selected food commodities in Bhutan

**DOI:** 10.1038/s41598-024-63677-6

**Published:** 2024-07-15

**Authors:** Adeep Monger, Pooja Mongar, Tshering Dorji, Vishal Chhetri

**Affiliations:** Royal Centers for Disease Control, Ministry of Health, Thimphu, Bhutan

**Keywords:** Aflatoxins B_1_, Bhutan, Risk assessment, Total Aflatoxins, Microbiology, Environmental sciences, Health care, Risk factors

## Abstract

Aflatoxins are mycotoxins that contaminate staple foods globally and pose a significant health risk. To the best of our knowledge, information on the occurrence of aflatoxins in Bhutanese diets is scarce. This study aimed to estimate the aflatoxin levels in selected foodstuffs in Bhutan and determine the health risk associated with aflatoxin exposure. Ten different types of food commodities were randomly collected from farmers’ markets, shelves of supermarkets, and wholesale and retail shops from 20 districts of the country. The samples were subjected to analysis by an enzyme-linked immunosorbent assay for both total aflatoxins (B_1_, B_2_, G_1_ and G_2)_ and aflatoxin B_1_. Among the 315 samples included, 48.81% and 79.35% were positive for total aflatoxins and aflatoxin B_1_, respectively. The overall mean total aflatoxin concentration was 11.49 ± 12.83 µg/kg, and that for B_1_ was 17.62 ± 23.99 µg/kg. The most prevalent food commodity with the highest aflatoxin contamination was chili products. In addition, the estimated daily intake and margin of exposure to aflatoxin B_1_ via the consumption of chili products ranged from 0.98 to 5.34 ng kg^−1^ bw day^−1^ and from 74.90 to 408.10, indicating a risk for public health. The liver cancer risk was estimated to be 0.01 and 0.007 cancers per year per 100,000 population resulting from the consumption of chili products. The present findings revealed the presence of total aflatoxins and aflatoxin B_1_ in the selected samples. The margin of exposure values was exorbitant, demanding a stringent public health measure. Notably, these results suggest the need for routine monitoring of aflatoxin contamination in the region and stress rigorous safety management strategies to reduce exposure.

## Introduction

Aflatoxins are secondary metabolites produced by two related species of fungi, *Aspergillus flavus* and *Aspergillus parasiticus*^1^. These toxins contaminate many staple foods and are a significant concern for public health^2,3^. Several types of aflatoxins occur in nature, and the four major aflatoxins are AFB_1_, AFB_2_, AFG_1_ and AFG_2_, which are unsafe for humans and animals^4^.

Staple dietary foods such as maize, groundnuts, rice and milk are highly susceptible to aflatoxin contamination, making them a real threat to food security, safety and population health^5,6^. As a result, exposure to aflatoxins via the diet is associated with various health consequences^7,8^. The ingested aflatoxins are converted to aflatoxin-8,9-epoxide metabolites in the liver, which are responsible for many of the toxic effects in the body^9^. Acute toxicity includes hemorrhage, acute liver damage, edema, digestion problems and can sometimes precede death^10^, whereas chronic exposure leads to the alteration of intestinal integrity, resulting in stunting in children and immune suppression^11,12^. The International Agency for Research in Cancer classified AFB_1_ and mixtures of total aflatoxins as group I carcinogens that induce liver and colo-rectal cancer^13^. Notably, these implications are frequently reported in regions where the prevalence of aflatoxin occurrence is high^3^.

Aflatoxin contamination poses a global threat, as an estimated 25% or more of the world’s food crops are destroyed annually, posing a significant economic burden^14^. Warm and humid storage conditions allow the growth of molds, and aflatoxins accumulate in commodities throughout any stage of the value chain from preharvest to post harvest^15^. The incidence of aflatoxin contamination is reportedly highest in developing countries and, more specifically, in Asia^16^. This is due to their geographical location, as it provides favorable conditions for the proliferation of mycotoxins and the implementation of poor regulatory measures, resulting in potential exposure of more than 5 billion human populations^17^. For instance, in a study conducted by Jog Raj in 2021, 93% of the tested corn samples collected from India, Thailand, Vietnam, Taiwan and the Philippines were contaminated with one or multiple aflatoxins^18^.

To date, no study has been conducted on the contamination levels of food commodities by aflatoxins in Bhutan. Considering the country’s warm and humid climate due to global warming, low income and poor stringent rules and regulations, it is of paramount importance that the prevalence of aflatoxin contamination in major staple foods in the country be investigated.

Furthermore, it is important to conduct risk assessments because the complete elimination of aflatoxins is not possible. Risk assessment is the process of characterizing the potential hazards and associated risks resulting from exposure to chemicals present in food over a specific period^19^. This approach is used to guide food regulators and public health officials in implementing risk mitigation processes, such as setting regulatory limits on food supplies. Although Bhutan has set the maximum tolerable concentration of total aflatoxins to 20 µg/kg, no risk assessment has been conducted to date to evaluate the potential health implications of aflatoxin exposure. This is essential due to differences in dietary habits, exposure and characterization of the risk it possesses. Therefore, considering the scarcity of information on aflatoxin contamination in the region, the present study attempted to determine the occurrence of aflatoxin contamination in different food items in Bhutan, and a deterministic risk assessment was performed based on dietary exposure to aflatoxins.

## Results

### Validation of the analytical method

The calibration curve plotted using standard log concentration versus Logit B/Bo by linear regression showed excellent linearity, with a correlation coefficient > 0.999 for both total aflatoxin (AFT) and aflatoxin B_1_ (AFB_1_). The relative standard deviation of all the spiked samples ranged from 1.66 to 6.95%, and the recoveries of both AFT and AFB_1_ ranged from 87.19 to 112.02%. Furthermore, the limit of detection was adopted according to the manufacturer’s values of < 3 µg/kg for AFT and < 2 µg/kg for AFB_1_. Table 1 illustrates the findings.

**Table 1:** Total aflatoxin and aflatoxin B_1_ recoveries.

Mycotoxin types	Spiked (µg/kg)	Recovery (%)	RSD (%)
Total aflatoxin	4	87.19	5.30
	40	91.21	4.71
Aflatoxin B_1_	20	112.02	1.66
	50	96.40	6.95

### Occurrence of aflatoxins

A total of 315 samples consisting of 10 different categories of food commodities were analyzed for the presence of AFT (B_1_, B_2_, G_1_ and G_2_), and those samples detected with > LOD of AFT were further subjected to analysis for aflatoxin B_1_ (Table 2). Of these, 48.81% of the samples were positive for AFT, and 79.35% were positive for AFB_1_. The mean levels of AFT and B_1_ in all the samples were 11.49 ± 12.83 µg/kg and 17.62 ± 23.99 µg/kg, respectively. The concentrations of AFT in the 95th and 50th percentiles were 41.98 and 6.84 µg/kg, respectively, while those of AFB_1_ were 85.89 and 10.21 µg/kg, respectively.

**Table 2:** Levels of total aflatoxins and aflatoxin B_1_.

Mycotoxin type	n	Mean ± SD	50th percentile	95th percentile	Samples < LOD	Positive frequency (%)
Total aflatoxin	315	11.49 ± 12.83	6.84	41.98	161	48.81
Aflatoxin B_1_	155	17.62 ± 23.99	10.21	85.89	32	79.35

Table 3 illustrates the results of the analyzed samples based upon the type of sample and the occurrence and concentration of aflatoxins. The overall concentrations of AFT and AFB_1_ ranged from less than the limit of detection to 75.84 µg/kg and 116.41 µg/kg, respectively. Among the types of food samples, chili powder had the highest mean level of total aflatoxin (19.65 µg/kg), followed by nuts (14.06 µg/kg). Similarly, chili powder was the dominant food commodity, with the highest mean concentration of aflatoxin B_1_ detected (28.01 µg/kg), followed by nuts (22.96 µg/kg). Although split pulses had a higher mean, only one sample was detected with a high level of B_1_, which resulted in a higher overall mean. Notably, none of the rice samples tested had > LODs for AFT; hence, these samples were not subjected to further testing for AFB_1_.

**Table 3:** Levels of AFT and AFB_1_ in different food commodities in Bhutan.

Food samples	Total aflatoxin					Aflatoxin B_1_				
	n	Mean	SD	Min	Max	n	Mean	SD	Min	Max
Chilli powder	39	19.65	16.32	< LOD*	75.84	39	28.01	26.84	5.47	116.41
Dried chilli	18	9.96	2.59	< LOD*	13.69	15	11.18	4.21	6.06	21.67
Nuts (peanut, chasew nut)	49	14.06	12.95	< LOD*	59.03	26	22.96	29.39	< LOD**	102.7
Corn products (Tengma, Kharang)	48	4.48	0.86	< LOD*	5.69	11	6.55	5.51	< LOD**	10.45
Flour products (Kabchi, Nabchi)	24	7.79	6.51	< LOD*	22.74	15	5.58	5.19	2.24	18.33
Rice and rice products (flattened rice)	21	5.27	NA	< LOD*	5.27	0	–	–	–	–
Dried fish	40	8.12	11.7	< LOD*	62.28	24	8.75	21.28	2.06	95.74
Dried beef	13	5.51	1.82	< LOD*	8.96	6	3.68	1.22	< LOD**	5.08
Split pulses (peas, lentils)	39	4.18	0.94	< LOD*	6.56	12	44.83	60.19	< LOD**	87.39
Soya chunk	20	6.27	2.44	< LOD*	10.56	9	9.56	7.05	2.13	20.49

The *LOD for total aflatoxin = 3 µg/kg, **LOD for aflatoxin B_1_ = 2 µg/kg. (Tengma (beated corn), Kharang (ground corn granules)). Kabchi (wheat flour), Nabchi (millet flour)).

The concentration of chili products was significantly greater than that of all other food items (p value = 0.00104). Moreover, the concentration of aflatoxin B_1_ correlated well with the AFT concentration, ranging highest in chili powder, nuts and dried chili, at 27.42 ± 26.49, 23.41 ± 30.82 and 11.41 ± 4.27 µg/kg, respectively.

**Figure 1:**
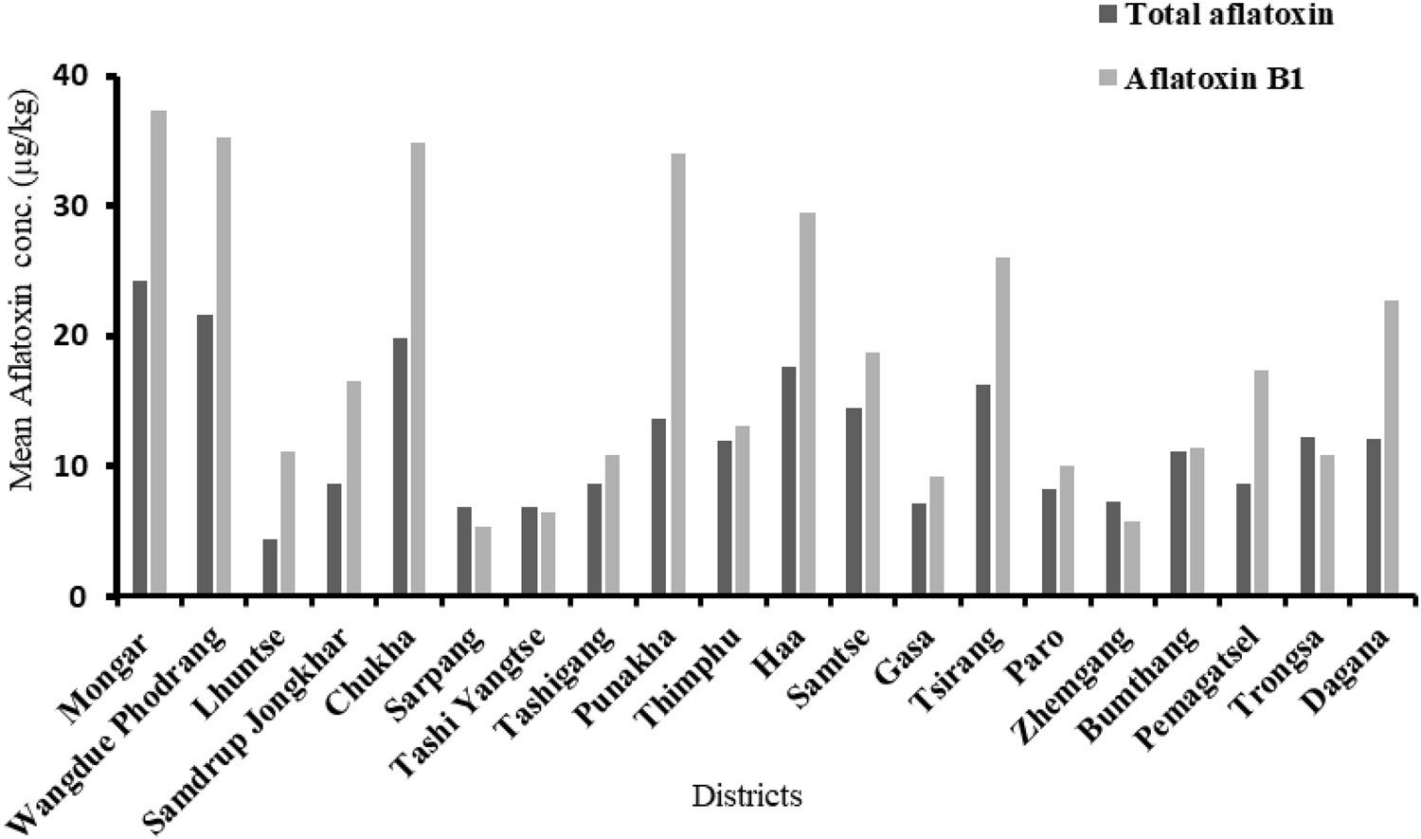
Burden of aflatoxins in different districts of Bhutan.

With respect to the mean AFT concentration in the food samples from the different districts, the highest concentration was detected in Monggar (24.19 µg/kg), followed by Wangdue Phodrang (21.7 µg/kg) and Chukkha (19.88 µg/kg). The lowest values were determined for the districts of Lhuentse, Sarpang and Trashiyantse. Similarly, aflatoxin B_1_ was also found to be the most abundant toxin in the abovementioned districts, at 37.30, 35.31 and 34.85 µg/kg, and the least abundant were from Sarpang, Tashiyantse and Gasa. However, there was no significant difference in the concentration of aflatoxins in the food samples (p value = 0.03–0.64). Figure 1 illustrates the findings.

Bhutan is divided into four regions and there is a difference in culture, food habits, ethnicity and climatic conditions. Hence, an attempt was made to report aflatoxin contamination in different regions of the country. The supplementary table shows the different levels of total aflatoxins detected in different regions of the country (see Supplementary Table S1). The highest percentage of positive samples was detected in the western region (56.84%), followed by the central region (56.67%). However, the southern region had the highest mean aflatoxin concentration (14.76 ± 16.15 µg kg^−1^), followed by the western region (12.93 ± 15.57 µg kg^−1^). One-way ANOVA was performed to compare the means of these different regions; however, no significant differences were detected in the mean level of aflatoxins.

The results from the analyzed samples were further compared with international guidelines elsewhere (CODEX, the EU and the US FDA). In total, 43.6% of the total samples had total aflatoxin levels above the European Union maximum level of 4 µg/kg, whereas 9.1% and 4.6% of the samples had total aflatoxin levels above the CODEX and US FDA maximum limits, respectively (Table 4). To ensure maximum food safety, foodstuffs were more specifically compared with the EU regulations of minimal aflatoxin contamination. The results showed that the chili powder had the highest level of aflatoxins above the regulatory limit of 94.87%, followed by dry chili (77.7%) and peanuts (60.6%). Supplementary Table S2 shows the data.

**Table 4:** Proportion of samples above the maximum regulatory limits.

Sl. No	Organization	Maximum level (ML) µg/kg	Samples above the ML	
			Number (n)	Frequency (%)
1	CODEX	15.0	28	9.1
2	EU Regulations	4.0	133	43.6
3	US FDA	20.0	14	4.6

### Human health risk characterization

Human health risk estimation was conducted on the most consumed food items in the country, which were chili products (chili powder and dried chili). The results are illustrated in \* MERGEFORMAT Table 5. The exposure estimates were calculated based on the estimated daily intake (EDI) of these food items. In the present study, the daily intake of chili powder and dry chili was estimated to be 2.1 g/day and 15.92 g/day, respectively, for an adult weighing an average body weight of 60 kg, which was determined from the Bhutan Living Standard Survey 2022^20^. The EDIs for chili powder at the mean and 95th percentile exposure was 0.98 and 5.34 ng/kg^−1^ bw day^−1^, respectively. Similarly, for the dried chili, the mean exposure was 2.96, and for 95th percentile was 5.30 ng kg^−1^ bw day^−1^.

**Table 5:** Human health risk estimation due to aflatoxin B_1_ exposure in Bhutan.

Food samples		Daily intake (gram day^−1^)	Mean aflatoxin B_1_ conc. (ug/kg)	Exposure/EDI (ngkg^−1^ bw.day^−1^)	Margin of exposure	Annual HCC incidence (cancer.yea^−1^.1,00,000^−1^
Chilli powder	Mean	2.10	28.01	0.98	408.10	0.02656
	95th	3.71	86.50	5.34	74.90	0.1447
Dried chilli	Mean	15.92	11.18	2.96	135.10	0.08024
	95th	18.10	17.59	5.31	75.40	0.14368

POD of BMDL10 = 0.4 µg kg^−1^ bw day^-1^, average body weight (adult)—60 kg.

Risk characterization was conducted based on the margin of exposure (MOE) and the liver cancer approach. MOE compares exposure levels to a toxicological threshold and is typically used to compare the health risk of various contaminants to prioritize risk management efforts^21^. The lower the MOE, the greater the risk to the consumers and a value of less than 10,000 is used to indicate health risk^22^. This is measured by the benchmark dose lower confidence limit (BMDL) for a benchmark response of 10% of 0.4 µg/kg body weight per day for the incidence of hepatocellular carcinoma (HCC) in male rats following AFB_1_ exposure as per the European Food Safety Authority^23^.

The range of MOEs for BMDL_10_ derived from the analysis ranged from 74.90 to 408.10. From these calculations, the MOE values were less than 10,000, indicating a public health risk due to AFB_1_ exposure through the consumption of chili products. Remarkably, if the 95th percentile values for large consumers are considered, there is a significant risk from exposure to aflatoxins to the general population. The incidence of HCC was estimated by the quantitative cancer risk approach and is expressed as cancer cases per 100,000 individuals per year. The values show that, for the maximum exposure scenario, the cancer risk was estimated to be 0.01 cancer cases per 100,000 people per year for the Bhutanese population.

**Figure 2:**
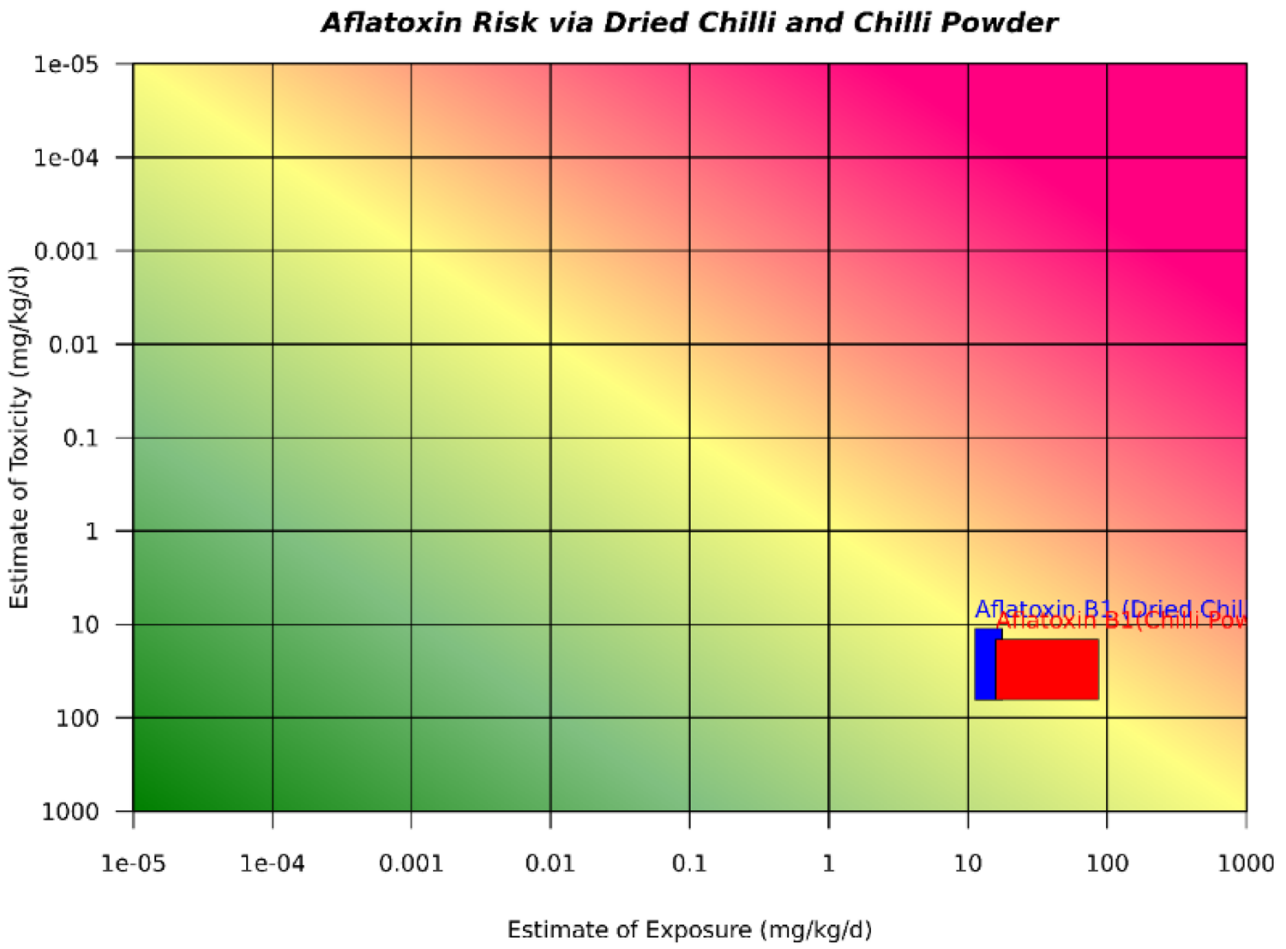
Risk 21 plot for chili products.

Figure 2 shows the plot for exposure to AFB_1_ and the estimate of toxicity due to consumption of dried chili and chili powder in the Bhutanese population. The intersection of the estimates of toxicity and the estimates of exposure revealed the risk of AFB_1_. A higher risk and toxicity of AFB_1_ are illustrated by the closer point of intersection to the x-axis and farther from the y-axis. According to the risk 21 matrix, chili powder has a greater risk of daily exposure to AFB_1_ than does dry chili.

## Discussion

This study reports the first findings on the status of aflatoxin contamination in major staple foods consumed in the region and its associated human health risk. The report revealed the presence of total aflatoxins and aflatoxin B_1_ in almost all of the samples included in the test. The MOE values demand a stringent public health measure.

An initial step for determining the true burden of aflatoxin contamination is the use of proper test assays^24^. For a specific test to be suitable for its intended use, the quality, reliability and consistency of the analytical results are integral. For this purpose, recovery analysis was performed to determine the actual concentration of an analyte in the samples, and we reported recoveries ranging from 87.19 to 112.02% for both AFT and AFB_1_. These percentages were within the acceptable range of the AOAC, CODEX and EU regulations of 60–120%^25^; therefore, the analytical method was reliable and in accordance with international guidelines.

The present study revealed a positive frequency of 48.85% for AFT and 79.35% for AFB_1_. This indicates that the Bhutanese population is exposed to the carcinogenic toxins that are present in the daily diet. Similar studies performed in Thailand reported an occurrence of 38.9%^26^; 27.1%, in Zhejiang Province, China^27^; 24.3%, in Vietnam^28^; 78.0%, in Nepal^29^; and 61.3%, in India^30^. The presence of these aflatoxins is inevitable, as factors such as changes in temperature and water availability related to actual climate changes (increased temperature, heavy rainfalls and droughts) all modulate mold growth and the production of mycotoxins^31^. This demands a safe and effective multifaceted approach for combating food and feed contamination. Although most developed countries have stringent rules for regularly monitoring these toxins in food, ensuring food insufficiency and security is a major challenge for developed and developing countries.

Studies across the region have reported AFB_1_ concentrations ranging from 48–383 µg/kg in corn samples in India, 0.04–21.30 µg/kg in rice in Pakistan and 1–34.80 µg/kg in corn in Vietnam^32^. In the present study, the overall AFT and AFB_1_ concentrations ranged from < LOD to 75.84 µg/kg to 116.4 µg/kg for all the food commodities. This is very alarming since getting exposed to such a level of toxins leads to severe health consequences. A study by Angele et al. revealed that a concentration of 0.099 µg/kg in eggs and 0.525 µg/L in milk are risk factors that could increase the incidence and prevalence of malnutrition and cancer in Cameroon^33^. A similar study in India by Asim et al. revealed a threefold increase in the risk of hepatocellular cancer with respect to urinary AFB_1_ and a higher incidence of aflatoxin contamination in the region^34^. Currently, 27.5% of the children in the country are stunted, almost half (42.6%) of the stunted children are severely shunted^35^, and the incidence of liver cancer is 5.3%^36^. The association between exposure to aflatoxins and the probability of stunting and the development of liver cancer should be considered. The question arises whether these health implications are attributed to issues related to the contamination of aflatoxin in the food that the population consumes daily. An exposure assessment study using appropriate biomarkers for aflatoxin exposure involving the recruitment of liver cancer patients and shunted children is necessary.

Notably, there was a clear difference in the concentrations of both AFT and AFB_1_ among the different districts of the country. This may be attributed to the geographical locations of the districts. For instance, the Monggar district, which falls in the eastern region of the country, had an overall mean concentration of 24.19 µg/kg, and the Chukkha district, which lies in the southern region, had 19.88 µg/kg. The climate conditions vary among these regions; thus, differences were observed. A study by Battilani et al. reported that in Europe, aflatoxin B_1_ contamination in maize has increased due to climate change^37^. This finding is further supported by the findings of Benigni et al. and Sarah et al., whose findings correlated well with the current findings^38,39^.

The most prevalent foods contaminated with aflatoxins reported in this study are chili products, followed by nuts. Contamination of chili powders by aflatoxins has been described by several surveys from studies in the region. In India, almost 66.7% of the samples had values above 10 µg/kg^40^; 86.7%, in Thailand^41^; and 100% of the samples > 20 µg/kg, in Bangladesh^42^. Similarly, in the present study, 84.6% of the chili powder samples had a concentration greater than 10 µg/kg, which is in concordance with the findings of other studies. Chili powder is produced locally using traditional methods by sun drying the chilies and grinding it to powder. In this process, there is a maximum likelihood of mold formation. The occurrence of aflatoxin contamination is determined by factors such as pre-harvest, post-harvest, storage conditions, temperature and humidity^15,32^. Therefore, prevention measures such as treating fungal contamination in crops, appropriately handling food items, ensuring effective food regulation systems and providing mass advice are essential for minimizing aflatoxin contamination in food.

The estimated daily intake of AFB_1_ via the consumption of chili products was determined to be 0.98 and 2.96 ng/kg^−1^ bw day^−1^ in the present study. Studies elsewhere in Ethiopia reported an EDI of 0.13–15.8 ng/kg^−1^ bw day^−1^^43^, and those in Malaysia were 0.21–1.32 ng/kg^−1^ bw day^−1^^44^. This discrepancy might be due to differences in the concentrations and consumption data collected during the particular study period. Nevertheless, the findings reveal that the risk of aflatoxin B_1_ exposure originates from both the high occurrence of aflatoxin B_1_ contamination and the high consumption of chili products by the population. Taken together, these findings indicate that the current regulatory limits of 20 µg/kg may still not be low enough to eliminate these concerns.

Based on the current findings, it is imperative that the intake of contaminated chili products is of great importance for public health since all the MOE values are below the safe threshold value of 10,000. The MOE values of AFB_1_ recorded in the present study ranged from 74.90–408.10, which are comparable to those reported for chili products from Sri Lanka ranging from 45–78 and 997–909 in Turkey^45,46^, exposing the respective consumers.

## Conclusion

This study illustrates the presence of total and aflatoxin B_1_ in selected food commodities from the country, providing the first insights into their occurrence and human health risk assessment. The incidence of both total and aflatoxin B_1_ contamination in food samples was greater than that in half of the samples. The margin of exposure was less than the safety limit of less than 10,000, indicating a serious public health concern. In addition, the liver cancer risk was estimated to be 0.01 and 0.007 cancers per year per 100,000 population resulting from the consumption of chili products. The data presented here provide evidence that addressing aflatoxin contamination and exposure to aflatoxins is necessary and may cause severe health consequences for the population.

## Methods

### Study area and site

**Figure 3:**
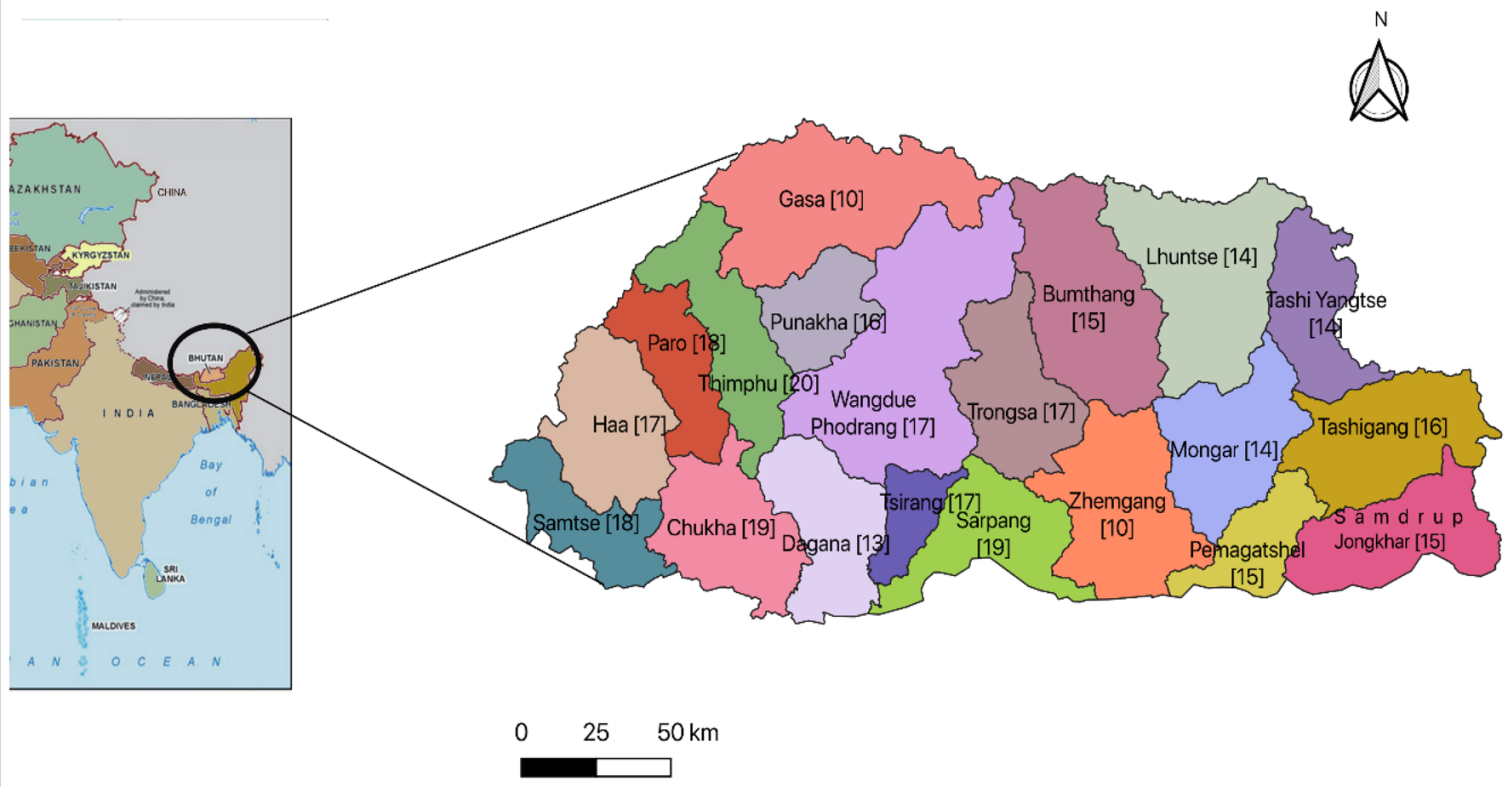
Sample collection sites. The map was created with qgis version 3.26 (https://www.qgis.org/).

The Bhutan region is a subtropical region and has twenty administrative districts. The study site was located in each district, and samples were collected from each site. Figure 3 represents the districts of the country and the number of samples collected from each district.

### Sampling

A total of 10 different categories of food commodities were randomly sampled and purchased between September and October 2022 from grocery and supermarket shelves. Each sample was homogenized by grinding, and an aliquot of 20 g was kept in zip lock bags and stored at − 20 °C until analysis. Based on the literature on the highest likelihood of aflatoxin contamination, the following types of samples were collected and subjected to chemical analysis (Table 6).

**Table 6:** Types of food commodities included in the study.

Sl No	Commodities
1	Chili powder
2	Dried chili
3	Nuts (peanut, chasew nut)
4	Corn products (beated corn (tengma), ground corn granules (kharang)
5	Flour products (Kabchi-wheat flour, Nabchi-millet flour)
6	Rice and rice products (flattened rice)
7	Dried fish
8	Dried Beef
9	Split pulses (peas, lentils)
10	Soya chunk

### Chemicals

Commercially available detection kits (AgraQuant, Romer labs, Inc., Newark, DE, USA) for both total aflatoxin (AFB_1_, AFB_2_, AFG_1_ and AFG_2_) and AFB_1_ were used for detection and quantification in the present study. The methanol used for extraction was of HPLC grade, and all the other chemicals used were of ACS grade or higher.

### Sample analysis

Briefly, 20 g of representative sample was meshed and extracted with 100 ml of 70% methanol. The solution was vortexed and settled, and the top aqueous layers were filtered using a Whatman No. 1 filter. The extracted samples were subsequently analyzed using direct competitive enzyme linked immuno-sorbent assay (ELISA) to determine the total aflatoxin AFB_1_, AFB_2_, AFG_1_ and AFG_2_ concentrations.

### Health risk estimation

#### Estimated daily intake

The estimated daily intake (EDI) of aflatoxin via oral ingestion was calculated by using the mean and 95^th^ percentile levels of total aflatoxins and aflatoxins B_1_ determined from the food samples and the daily intake by the population divided by the average body weight. The following equation was used for the calculation of the EDI:

$${\text{EDI(ng}}\,{\text{kg}}^{\text{-}1} {\text{day}}^{\text {-}1} {) = }\frac{{{\text{Daily}}\,{\text{Intake}} \times {\text{Mean}}\,{\text{Concentration}}}}{{{\text{Average}}\,{\text{Body}}\,{\text{Weight}}}}$$

#### Margin of Exposure (MOE)

For the derivation of the MOE, the point of departure or reference point from the animal data was divided by the estimated dietary exposure.

$$\text {MOE}=\frac {\text {BMDL}_{10}\; (\text {ng/kg.bw/day})} {\text {Estimated\,Daily\,Intake}\;(\text {ng/kg.bw/day})}$$

#### Cancer risk

To estimate the potency of aflatoxin B_1_, we adopted the proposed JECFA formula to evaluate the carcinogenic risk in humans. Therefore, 0.3 cancer/year/100,000 population ng/kg bw/day for HBsAg-positive individuals and 0.01 cancer/year/100,000 population ng/kg bw/day for HBsAg-negative individuals were considered to determine the average potency. Moreover, a HBsAg-positivity prevalence of 5.9% was adopted for Bhutan^47^, and the HBsAg-negative group was extrapolated to 92.26%. The following equation was used for the calculation:

$${\text{Average}}\,{\text{Potency}} = (0.3 \times {\text{HBsAg}}{-}{\text{ve}}\,{\text{prevalence}}) + (0.01 \times {\text{HBsAg}} + {\text{ve}}\,{\text{prevalence}})$$

$${\text{Cancer risk = Exposure/EDI}} \times {\text{Average potency }}$$

#### Data analysis

The concentrations of AFT and AFB_1_ were calculated via regression analysis from the standard calibration curves generated from standard reference materials of both AFT and AFB_1_ using the standard spreadsheet provided by the manufacturer (Romerlabs). The data are presented as the mean, standard deviation and 50th to 95th percentiles. The significance differences among the food sample types and regions were analyzed using ANOVA tests in SPSS version 22 (IBM, Chicago, IL, USA). For the risk assessment models, estimated daily intake, MOE values, average potency and cancer risk were all used as per the guidelines of the EFSA and WHO^23,48,49^.

### Ethical issues

No ethical approval was needed because there were no human participants involved. However, permission for a waiver of ethical clearance was sought from the Research Ethics Board of Health, Bhutan (Ref. No. REBH/PO/2022/029). Verbal informed consent was obtained from the respective shopkeepers and vendors during sample collection.

## Data Availability

All relevant data are within the paper and its supporting information files.
